# Copy number variations at the *Rhg1* locus and their relationship with resistance to soybean cyst nematode (*Heterodera glycines*)

**DOI:** 10.3389/fpls.2024.1504932

**Published:** 2024-12-18

**Authors:** Dinesh Poudel, Guiping Yan, Carrie Miranda, Gustavo Fernando Kreutz, Intiaz Amin Chowdhury

**Affiliations:** ^1^ Department of Plant Pathology, North Dakota State University, Fargo, ND, United States; ^2^ Department of Plant Sciences, North Dakota State University, Fargo, ND, United States

**Keywords:** soybean, soybean cyst nematode, HG type, Rhg1, copy number, female index

## Abstract

Soybean cyst nematode (SCN, *Heterodera glycines*) is a devastating pest affecting soybean production worldwide. Host resistance is one of the primary practices used to manage SCN. The *Rhg1* locus contributes to the strong and effective SCN resistance, with resistance levels predominantly governed by copy number variations (CNVs) and, to lesser extent, sequence variations. This study assessed the host resistance of 100 soybean breeding lines to SCN populations HG type 2.5.7 (S1) and HG type 7 (S2). Two controlled growth chamber experiments involved inoculating plants with 2,000 SCN eggs and juveniles, followed by counting SCN white females and calculating the female index (FI) to classify resistance responses. To determine CNVs at the *Rhg1*, a SYBR Green-based quantitative PCR (qPCR) assay was optimized and validated using 12 soybean accessions with known copy numbers. The qPCR assay demonstrated 94.36% efficiency for the target gene at *Rhg1* locus, *Glyma18g02590*, with copy number detected by the assay correlating strongly (*r=*0.994) with whole genome sequencing data in previous study. Copy number of each line was determined using 2^−ΔΔCq^ method relative to Williams 82 (single copy) and correlated with the resistance response. One line, ND20-16996(GT) was resistant (FI<10%) to S2 in both runs, while none were resistant to S1. Copy number among the breeding lines ranged from 1 to 11, with higher copy numbers correlating negatively with female index, indicating greater resistance. The breeding lines with copy number ≥ 9 were either resistant or moderately resistant to S2, and mostly moderately resistant to S1, with few being moderately susceptible (FI=30 to <60%). The lines with low copy numbers (≤3) were mostly susceptible (FI≥60%) to S1, while moderately susceptible or susceptible to S2. These results show the importance of *Rhg1* CNVs in determining levels of SCN resistance and selecting resistant soybean lines.

## 1 Introduction

Soybean cyst nematode (SCN; *Heterodera glycines*) is a devastating obligate endoparasitic plant nematode affecting soybean (*Glycine max* (L.) Merr.) production worldwide. In the United States, SCN has been the most significant yield-reducing factor causing an estimated $1.5 billion loss of revenue annually (Wrather and Koenning, 2009; Bandara et al., 2020). Following its first report in the USA in 1954 in North Carolina (Winstead et al., 1955), SCN has subsequently disseminated to almost all soybean-producing states (Tylka and Marett, 2021). Given the widespread and severe impact of SCN, effective management strategies are crucial to sustaining soybean production. Planting cultivars that are resistant to SCN and crop rotation with non-host crops are the common management tactics employed to control SCN (Niblack, 2005; Mitchum, 2016; Bent, 2022). SCN-resistant cultivars are particularly vital in regions where crop rotation alone is insufficient due to the nematode’s persistence in the soil for many years, even in the absence of a host. However, field populations of SCN exhibit significant diversity in virulence, limiting the effectiveness in specific regions with different SCN populations (Niblack et al., 2008; Acharya et al., 2016; McCarville et al., 2017; Chowdhury et al., 2021). The majority of commercially available soybean cultivars with resistance to SCN are derived from Plant Introduction (PI) 88788 and Peking (PI 548402) (Concibido et al., 2004). PI 88788 is by far the most widely used, and its extensive use can be attributed to its desirable agronomic characteristics, especially its capacity to uphold high yields (Kim et al., 2011). As SCN populations continue to evolve, screening soybean lines for resistance is important to identify lines effective against diverse SCN populations found in field conditions.

SCN variability is characterized by the term ‘HG type’ which is based on the number of SCN females that develop on seven specific PI soybean lines: PI 548402, PI 88788, PI 90763, PI 437654, PI 209332, PI 89772, and PI 548316, in comparison to the number of females formed on a susceptible soybean cultivar (Niblack et al., 2002). The female index, a comparative measure of the number of SCN females developing on these lines, is often used to assess the reproductive potential of SCN within a specific soybean line (Beeman et al., 2016). Variations in the female index can reflect differences in the virulence and adaptability of SCN populations (Beeman et al., 2016; Tylka, 2016). Previous studies have demonstrated that soybean cultivars exhibit varying resistance responses to different HG types, as indicated by differences in the female index (Koenning, 2004; Acharya et al., 2017). For instance, HG type 2.5.7 shows a greater ability to reproduce on PI 88788 and its derivative cultivars than HG type 7 (Mitchum et al., 2007; Hershman et al., 2008; Niblack et al., 2008; Chen et al., 2010; Faghihi et al., 2010; Acharya et al., 2016). Hence, screening soybean lines for their response to multiple HG types provides a holistic insight into the extent of resistance they offer against diverse SCN populations in the field. In this study, we have compared the resistance responses of breeding lines to two SCN populations, HG type 2.5.7 and HG type 7, shedding light on potential genetic mechanisms underlying resistance to different HG types.

Most SCN-resistant commercial cultivars predominantly rely on two loci for resistance: *Rhg1* (Resistance to *Heterodera glycines* 1) on chromosome 18 and *Rhg4* on chromosome 8 (Concibido et al., 1994, 1997; Chang et al., 1997; Cook et al., 2012; Liu et al., 2017; Huang et al., 2021). The *rhg1* gene is a recessive or partially recessive resistance gene, and has been integrated into various high-yielding soybean cultivars, providing strong resistance to SCN (Concibido et al., 1997, 2004; Brucker et al., 2005; Cook et al., 2012). It confers resistance by disrupting the formation of syncytium induced by SCN (Mitchum, 2016). The *rhg1* haplotype of PI 88788, designated as *rhg1-b*, is used in approximately 90% of SCN-resistant commercial soybean cultivars within the central United States (Cook et al., 2012). PI88788 type resistance relies on high copy number of *rhg1* gene for resistance, named the *rhg1-b* allele, while the Peking type resistance has low copy number of *rhg1* gene, called *rhg1-a* allele and requires additional *Rhg4* gene for resistance (Mitchum, 2016). The allelic variation at *Rhg1* locus that determines resistance to SCN is governed by both copy number variations (CNVs) and sequence variation, with CNVs having a dominant role (Lee et al., 2015, 2016). Various resistant soybean accessions show differences in copy number and sequence of the repeats at the *Rhg1* locus (Cook et al., 2014; Lee et al., 2015). PI 88788 contains nine copies of a 31.2 kb tandem repeats with four genes, among which the expression of three genes encoding an amino acid transporter (*Glyma18g02580*), an α-SNAP protein (*Glyma18g02590*), and a wound-inducible 12 protein (*Glyma18g02610*) were up-regulated upon SCN infection and contributed to SCN resistance, which was found upon silencing the genes at the *Rhg1* locus (Cook et al., 2012). In contrast to PI 88788 and its derivative lines, susceptible lines exhibit a low copy number of the repeats (Cook et al., 2012, 2014; Lee et al., 2016; Shaibu et al., 2020). Therefore, analyzing the diversity at the *Rhg1* locus could help determine the extent of SCN resistance.

Various techniques are used to identify the copy number variations (CNVs), including array-based methods, such as comparative genomic hybridization (aCGH) (Debolt, 2010; Yu et al., 2011; Muñoz-Amatriaín et al., 2013), and single nucleotide polymorphisms (SNP) array (Cooper et al., 2008; Ma et al., 2017). aCGH compares fluorescence ratios between labeled test and reference DNA hybridized to genomic probes, while SNP arrays analyze signal intensity differences at specific SNP loci (Ylstra et al., 2006; LaFramboise, 2009). Both methods face challenges, including low resolution, sparse probe coverage, signal-to-noise issues, and susceptibility to false positives and negatives, requiring robust algorithms and validation for accurate detection (Carter, 2007). Shotgun sequencing fragments the genome into short reads, detecting CNVs through variations in read depth (Xie and Tammi, 2009). However, this method is not inherently quantitative and is limited by difficulty in accurately assembling regions with multiple copies, and reduced sensitivity at low coverage (Tammi et al., 2003). Whole genome sequencing (WGS) has been used in many studies to detect CNVs (Cook et al., 2012, 2014; Lee et al., 2015; Hu et al., 2018). Although this method identifies both SNPs and structural variants, it has limitations due to short reads and assembly difficulty (Gabrielaite et al., 2021; Coutelier et al., 2022). Next generation sequencing (NGS) provides tools for CNVs detection, however, it is expensive and requires appropriate statistical approach, specific bioinformatics tools, and algorithms, and often needs to be integrated with array based approaches and validated using quantitative methods for accuracy (Carson et al., 2006; Zhao et al., 2013; Wang et al., 2014; Gabrielaite et al., 2021). In contrast, quantitative PCR (qPCR) is a rapid, cost-effective, and precise method for CNVs detection at targeted loci and used in many studies (Li et al., 2004; Maron et al., 2013; Lee et al., 2015, 2016; Würschum et al., 2015; Huang et al., 2021). This method detects CNVs by comparing the threshold cycles (Ct) of a target gene with a reference sequence of normal copy numbers for CNV detection. It is widely used in large-scale studies to identify disease associations, validate computationally identified CNV loci, high-throughput screening of large cohorts of samples, and confirm regions identified in whole-genome scans using array-based methods (Carson et al., 2006; Li and Olivier, 2013). Recent researches have focused on modifying and optimizing the previously developed qPCR assays for effective CNVs detection at the *Rhg1* locus in soybean (Lee et al., 2015, 2016; Patil et al., 2019; Huang et al., 2021). Through the optimization of the qPCR technique, our goal is to enhance the efficiency and accuracy of CNV analysis at the *Rhg1* locus. Our approach involves the adoption, optimization, and validation of the real-time qPCR technique as reliable and accurate method for detecting CNVs in soybeans.

In this study, we screened 100 new soybean breeding lines under a controlled growth chamber condition to assess their resistance response against two prevalent SCN populations, HG type 7 and HG type 2.5.7 occurring in North Dakota. Additionally, we adopted, optimized and validated a real-time qPCR assay to quantify CNVs at the *Rhg1* locus, and explain the potential associations between CNVs and the resistance response exhibited by the breeding lines to SCN. Our findings elucidate the differential susceptibility of these breeding lines to varying SCN populations, informing future breeding efforts for enhanced resistance. Findings of this research can directly help in ascertaining extent of SCN resistance based on copy number variations at the *Rhg1* locus.

## 2 Materials and methods

### 2.1 SCN population characterization

Two previously collected and characterized populations of SCN, HG type 2.5.7 (population S1) from Richland County and HG type 7 (population S2) from Traill County in North Dakota (Chowdhury et al., 2021) were maintained under controlled greenhouse conditions by rearing on the susceptible soybean cultivar, Barnes. The virulence phenotype of these SCN populations were confirmed through two runs of HG typing test, following the procedure by Niblack et al., 2002. The two SCN populations were selected based on their contrasting reproductive capacities on the PI 88788 soybean line. Population S1 had a female index over 10% in PI 88788, indicating high reproduction, while population S2 had a female index below 10%, indicating lower reproduction in PI 88788 (Niblack et al., 2002).

### 2.2 Plant materials

Seeds of 100 soybean breeding lines were obtained from the soybean breeding program at North Dakota State University (NDSU), North Dakota (ND), USA. NDSU breeding lines were selected for testing based on entry into intermediate and advanced yield trials. The chosen breeding lines have not undergone prior evaluation for their resistance reactions to HG type 2.5.7 and HG type 7 and parental contribution of SCN resistance is unknown. Four plant introduction lines (PI 548408, PI 88788, PI 209332 and PI 548316) were used as controls, with Barnes (NDSU) serving as a susceptible check in the screening experiment. Additionally, 7 PI soybean lines were used for HG typing, and 12 soybean accessions (United States Department of Agriculture Soybean Germplasm Collection Center, Illinois) with known copy number at *Rhg1* locus, were used for validation of the qPCR assay.

### 2.3 Resistance response evaluation

Established protocols of Krusberg et al. (1994) were followed to extract SCN white females from greenhouse cultures for the inoculum preparation. SCN white females were gently crushed using a motor-driven rubber stopper mounted on a 250-μm sieve and SCN eggs and juveniles were collected on a 20-μm sieve stacked below 75-μm sieve (Faghihi and Ferris, 2000).

Seeds of each breeding line, control, and susceptible check, were pre-germinated in a petri-dish at normal room temperature for five days. Cone-tainers (3.8 cm wide, 21 cm tall; Stuewe and Sons, Tangent, OR) were filled with 100 cm^3^ of pasteurized river sand and arranged in a completely randomized design (CRD) with four replicates. Holes were made in the sand to accommodate seedlings of similar length, with each seedling from a specific soybean line planted individually in its designated hole. At the time of planting, each of the breeding lines, plant introduction lines, and susceptible check was inoculated with a 4 ml suspension containing 2,000 SCN eggs and juveniles, applied directly into the planting hole. The plants were then grown in a controlled growth chamber at a temperature of 27°C and a 16-hour daylight period, and daily watering was adjusted according to the growth stage of the plants. The plants were harvested 32 days after inoculation, and SCN white females were extracted from both the roots and soil of each pot together, following the standard protocol described by Krusberg et al. (1994).

Subsequently, the white females were counted on a lined petri-plate under a dissecting microscope (SM 100 Series, Swift Optical Instruments, Schertz, TX), and the counts across the four replicates were averaged to obtain the mean number of white females. This value was then utilized to compute the Female Index (FI) according to the formula (Acharya and Yan, 2022):


$$\text{FI \%}=\frac{\text{mean number of white females produced on the tested soybean line }}{\text{mean number of white females on the susceptible check},\text{ Barnes}}\times 100\%$$

Based on the FI values, soybean breeding lines were classified for their resistance responses, as described by Schmitt and Shannon (1992), into four categories: resistant (R) (FI<10%), moderately resistant (MR) (FI= 10 to <30%), moderately susceptible (MS) (FI= 30 to <60%), or susceptible (S) (FI ≥ 60%). The entire experiment was replicated once under similar controlled growth chamber conditions to ensure the reliability of the results.

### 2.4 qPCR assay for copy number assessment

All soybean lines were planted separately from the SCN screening experiment in greenhouse potting mix soil for tissue sampling. Genomic DNA was extracted from leaf tissue 10 days after planting using the FastDNA^®^ Spin Kit (MP Biomedicals, Santa Ana, CA) following the manufacturer’s manual. The genomic DNA was diluted to an average DNA concentration of 25 ng μl^-1^, and quantified using a NanoDrop^®^ ND-1000 UV-Vis Spectrophotometer (NanoDrop Technologies, Inc., Wilmington, DE).

To determine copy number at *Rhg1* locus, a genomic qPCR assay was adopted from Lee et al. (2015) and optimized. Forward primer 2590-F (5’-TGGAGTGGGCTGAATCTCTT-3’) and reverse primer 2590-R (5’-ATGGAAGCAAGAGCAGCATT-3’), targeting the gene in the duplicated region at *Rhg1* locus, *Glyma18g02590* were originally designed by Lee et al. (2015) and used in this study. An endogenous control gene from soybean’s heat shock protein (*Hsp*) gene family was utilized for normalization of all DNA samples (Li et al., 2009; Xing et al., 2010; Lee et al., 2015). The specific primers for the *Hsp* control gene were: forward primer (*Hsp*-F: 5’-CAAACTTGACAAAGCCACAACTCT-3’) and reverse primer (*Hsp*-R: 5’-GGAGAAATTGGTGTCGTGGAA-3’) (Li et al., 2009; Lee et al., 2015). The real-time qPCR assay in this study used SYBR Green dye, and qPCR conditions were optimized in terms of primer concentration, annealing temperature and DNA template concentration. A range of primer concentrations (50nM, 100 nM, 150 nM, 200 nM, 250 nM, and 300 nM) were tested to determine the optimal concentration for efficient amplification. Annealing temperatures (56°C, 56.7°C, 58.1°C, 60°C, 62.4°C, 64.4°C, 65.5°C and 66°C) were evaluated using gradient PCR approach to determine the optimal temperature for specific amplification. DNA template amounts (0.5 μl, 1 μl, 1.5 μl and 2 μl) were tested to find the optimal quantity for best results. All conditions were tested in triplicate, using DNA extracted from leaves of the Williams 82 soybean line as the template. The qPCR reaction was performed in 96-well plates using the Bio-Rad CFX96 Touch Real-time PCR Detection System (Bio-Rad Laboratories, Inc., Hercules, CA). All three technical replicates were run on the same plate for both the target and endogenous control gene. Each 10 μl qPCR reaction consisted of 5 μl of 2× SsoAdvanced™ SYBR^®^ Mastermix, and varying amounts of forward primer, reverse primer and nuclease-free H_2_O. The reaction was carried out using an amplification program consisting of an initial denaturation step at 95°C for 5 minutes, followed by 40 cycles of denaturation at 95°C for 30 seconds and varying annealing temperature for 1 minute. Melting curve profiles were generated by gradually increasing the temperature from 60 to 95°C, with increments of 0.1°C per 0.4 to 0.5 fluorescence units. Based on the analysis of amplification curves, quantification cycle (Cq) values, and melting curves, optimal primer concentration, annealing temperature and DNA template concentration were selected.

A set of 12 distinct soybean accessions, each characterized by a known number of *Rhg1* repeat copies (Lee et al., 2015) ranging from 1 to 10, were used as controls to validate the qPCR assay. Among the 12 soybean accessions used for validation, the genomic DNA from Williams 82, with a single copy of the *Rhg1* repeat, (Cook et al., 2012) was used as a reference. The relative copy number (RCN) of the repeat for other DNA samples were determined using the 2^(-ΔΔCq)^ technique, which calculates relative gene expression by comparing Cq values of the target and reference genes between experimental and control samples to determine fold changes (Livak and Schmittgen, 2001). The maximum and minimum bounds of RCN was determined by the formula of RCN_max_=2^−ΔΔCq+t*SEM ΔΔCq^ and RCN_min_=2^− ΔΔCq-t*SEM ΔΔCq^, where ‘t’ refers to the critical value from the t-distribution and ‘SEM’ refers to the standard error of the mean (Weaver et al., 2010). All the values for RCN were rounded up to the nearest integer. The amplification efficiency (E) was calculated by using the formula, E = 10^(1/–m)^-1, where ‘m’ corresponds to the slope of the standard curve generated through plotting Cq values against the logarithm of dilution of Williams 82 DNA, determined through sequential two-fold dilution series. The copy numbers derived from the qPCR assay for the 11 known copy number accessions were compared with the standard values from whole genome sequencing as reported by Lee et al. (2015), and a correlation analysis was done. After validation, the qPCR assay was subsequently deployed to investigate copy number variations within the *Rhg1* locus across the set of 100 breeding lines.

### 2.5 Data analysis

Data analysis was done using SAS 9.4 (SAS Institute, Cary, NC). Analysis of variance was conducted to access the effects of breeding line, HG type, experimental run and their interactions on the number of white females formed on the roots in the phenotypic screening for SCN resistance. For the copy number, 95% confidence intervals were calculated and represented as error bars. Correlation analysis was conducted to determine the associations between copy numbers and female indexes across both SCN populations and in both experimental runs.

## 3 Results

### 3.1 SCN population characterization

The HG type testing confirmed that the two SCN populations, S1 and S2, used as inoculum sources were HG type 2.5.7 and 7, respectively. The HG type tests were repeated for validation purposes and consistent results were observed for both experimental runs. HG type tests showed notable differences in the reproductive capabilities of the two nematode populations across the three primary plant introduction lines PI 88788 (#2), PI 209332 (#5), and PI 548316 (#7) (**Table 1**). Population S1 exhibited susceptibility in PI 88788, PI 209332, and PI 548316, with FI values consistently above 10% in both runs (**Table 1**). In contrast, Population S2 shows susceptibility only in PI 548316, with FI values above 10% in both runs (**Table 1**).

**Table 1:** HG type test results to confirm the two SCN populations, S1 and S2 used in this study.

	Plant Introductions (PIs)	Female Index (%)			
		S1		S2	
		Run 1	Run 2	Run 1	Run 2
#1	PI 548402 (Peking)	3.6	3.9	3.1	3.7
#2	PI 88788	25.2	25.6	7.2	8.8
#3	PI 90763	0.0	0.0	0.0	0.0
#4	PI 437654	0.0	0.0	0.0	0.0
#5	PI 209332	23.5	22.9	7.9	9.2
#6	PI 89772	0.0	0.0	0.0	0.0
#7	PI 548316	15.2	17.1	28.6	29.4
	HG type determination	HG type 2.5.7		HG type 7	

HG type for each of the SCN populations was determined using a female index (FI) threshold of 10% across seven plant introductions (PIs) in two experimental runs, following the standard procedures described by Niblack et al. (2002). Barnes was used as a susceptible check in both runs for both SCN populations. The average number of white females in the susceptible check (Barnes) were 353 and 350 for population S1, and 344 and 386 for population S2, for run1 and run 2, respectively.

### 3.2 Resistance response of soybean breeding lines

The effect of breeding line, HG type, experimental run and their interaction on the numbers of white females formed on the roots of the breeding lines were significant (*P*<0.001) (**Supplementary Table S1**). The four selected plant introductions, which served as controls, exhibited female index values consistent with those obtained in the HG-type test (**Table 1**; **Supplementary Table S2**). In both experimental runs, good reproductive response of the SCN was evident with the susceptible check (Barnes), where the average population of white females was 303 and 416 for population S2, and 279 and 431 for population S1, for run 1 and run 2, respectively.

**Figure 1:**
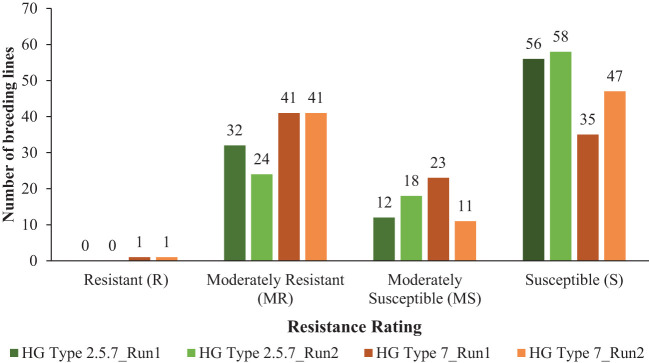
Distribution of 100 soybean breeding lines across four resistance categories: resistant (R) (FI < 10%), moderately resistant (MR) (FI = 10 to <30%), moderately susceptible (MS) (FI = 30 to <60%), and susceptible (S) (FI ≥ 60%), based on the criteria established by Schmitt and Shannon (1992) for soybean cyst nematode HG types 2.5.7 (S1) and 7 (S2). The number of breeding lines under each category screened for HG type 2.5.7 are represented by dark green and light green bars for run 1 and run 2, respectively, while those screened for HG type 7 are represented by dark brown and light brown bars for run 1 and run 2, respectively. The experiments were conducted under controlled growth chamber conditions with a temperature of 27°C and a 16-hour daylight period, across two experimental runs.

Among 100 breeding lines screened for population S1, 32 lines were moderately resistant (MR), 12 lines were moderately susceptible (MS) and the remaining 56 lines were susceptible (S) in the first run (**Figure 1**). In the second run, 24 lines were moderately resistant (MR), 18 lines were moderately susceptible (MS) and the remaining 58 lines were classified as susceptible (S) (**Figure 1**). Minor variations in female index values between the experimental runs led to shifts in the resistance responses for some lines screened against population S1. The female index ranged from 16.1-29.9%, 30.8-58.7% and 62.2-117.9% for moderately resistant, moderately susceptible and susceptible lines, respectively for population S1 in first run (**Supplementary Table S3**). In second run, female index ranged from 24.4-29.7%, 30.4-35.5%, and 60.6-93.2% for moderately resistant, moderately susceptible and susceptible lines, respectively (**Supplementary Table S3**). None of these breeding lines were resistant (R) to population S1 in the two experimental runs (**Figure 1**).

For population S2, one line, ND20-16996(GT), was resistant (R) in both runs, with female index of 7.0% and 9.7% in run 1 and run 2, respectively (**Supplementary Table S3**). In both runs, 41 lines were moderately resistant (MR) with FI values ranging from 10.5% to 25.9% in first run and 11.6% to 26.4% in second run (**Figure 1**; **Supplementary Table S3**). Twenty-three lines were moderately susceptible (MS) with FI values spanning from 41.6% to 59.7% and the remaining 35 lines were susceptible (S) with FI values from 60.1% to 143.8% in the first run (**Figure 1**; **Supplementary Table S3**). In the second run, 11 lines were moderately susceptible (MS) with FI values ranging from 46.8% to 58.8% and the remaining 47 lines were susceptible (S) with FI values spanning from 60.2% to 102.7% (**Figure 1**; **Supplementary Table S3**).

### 3.3 Optimization and validation of qPCR assay

Optimization of the qPCR conditions for detecting CNVs at the *Rhg1* locus in soybean identified optimal conditions as 200 nM primer concentration, 60°C annealing temperature, and 1.5 μl DNA template, which together yielded efficient and specific amplification. Amplification curves demonstrated clear exponential phases for both *Glyma18g02590* gene and *Hsp* gene, indicating efficient amplification (**Supplementary Figures S1A, C**). Consistent quantification cycle (Cq) values across technical replicates further supported the reliability of the qPCR conditions. Melting curve analysis revealed sharp, single peaks with no evidence of nonspecific amplification or primer-dimer formation, demonstrating the specificity of the reaction (**Supplementary Figures S1B, D**).

**Figure 2:**
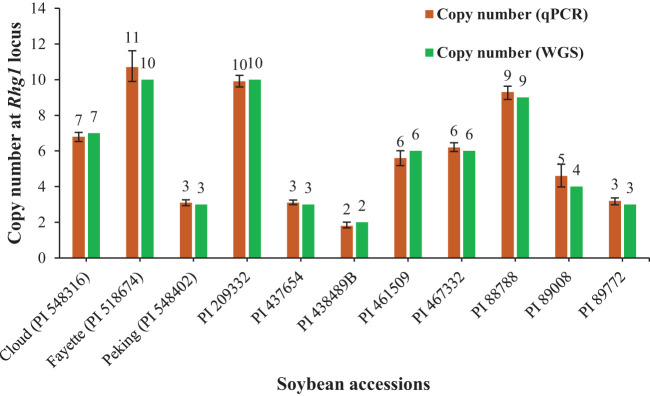
Distribution of copy number at the *Rhg1* locus in 11 soybean accessions used for qPCR assay validation based on the reference, Williams 82 with single copy number. The green bar represents the copy number determined by whole genome sequencing (WGS) in the reference paper: Lee et al. (2015) and brown bar represents the copy number detected by the qPCR assay targeting *Glyma18g02590* gene. Error bars, calculated at a 95% confidence interval, represent the upper and lower bounds of the copy numbers detected via the qPCR assay.

**Figure 3:**
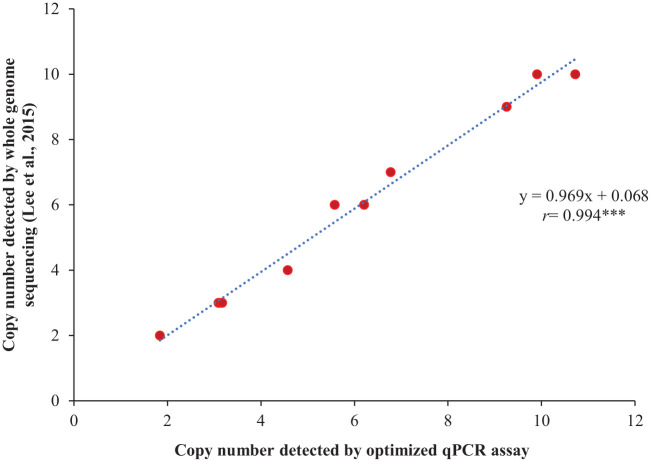
Validation of the qPCR assay through correlation analysis between the copy numbers of 11 soybean accessions, as detected by the optimized qPCR assay and those determined by whole genome sequencing in the study reported by Lee et al. (2015). The selected soybean accessions include PI 548316, PI 518674, PI 548402, PI 209332, PI 437654, PI 438489B, PI 461509, PI 467332, PI 88788, PI 89008, and PI 89772. Williams 82, with a single copy number at *Rhg1* locus, served as the reference for the qPCR assay. ‘*r’* represents Pearson’s correlation coefficient. *** indicates significant at *P*< 0.001.

**Figure 4:**
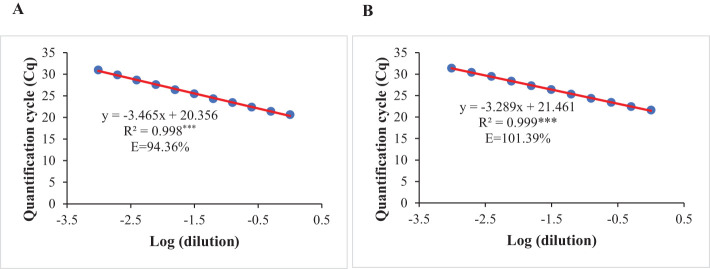
Standard curves prepared by plotting the quantification cycle (Cq) against the respective log dilution of genomic DNA extracted from leaves of Williams 82 for: **(A)** Target gene at *Rhg1* locus, *Glyma18g02590.* **(B)** Endogenous control, *Hsp* gene. Amplification efficiency (E) for each gene was calculated using the formula E= 10^1/–m^ – 1, where m is the slope of the respective standard equation. R^2^ represents coefficient of determination. *** indicates significant at *P*< 0.001.

The optimized qPCR assay determined the minimum and maximum bound of relative copy number, showing no significant difference compared to copy numbers determined by whole genome sequencing in Lee et al. (2015), thereby confirming the accuracy and reliability of the qPCR assay results (**Figure 2**). The high degree of correlation (*r*=0.994) between the copy number detected by the optimized qPCR assay and copy number determined by whole genome sequencing in Lee et al. (2015) underscores the assay’s precision in quantifying CNVs (**Figure 3**). Moreover, the standard curve generated from the data obtained with serial 2-fold dilution of genomic DNA of Williams 82 revealed a high degree of correlation between the Cq values and log_10_ values of dilution. For the target *Glyma18g02590* gene, a strong linear relationship was observed (R² = 0.998) with an efficiency of 94.36%, while the reference *Hsp* gene exhibited an even higher correlation (R² = 0.999) and efficiency of 101.39%, indicating the assay’s robustness and precision (**Figures 4A, B**). An amplification reaction of the DNA samples for target *Glyma18g02590* and control *Hsp* gene, including the Cq values are displayed by an amplification curve, where no amplification was observed in the control reactions as indicated by a straight curve below the threshold (**Supplementary Figures S1A, C**). Melting curve analysis revealed a single melting peak at 82°C and 76.5°C for the target gene (**Supplementary Figure S1B**) and control *Hsp* gene (**Supplementary Figure S1D**), respectively, confirming specific amplification.

### 3.4 Copy number variations at *Rhg1* locus

**Figure 5:**
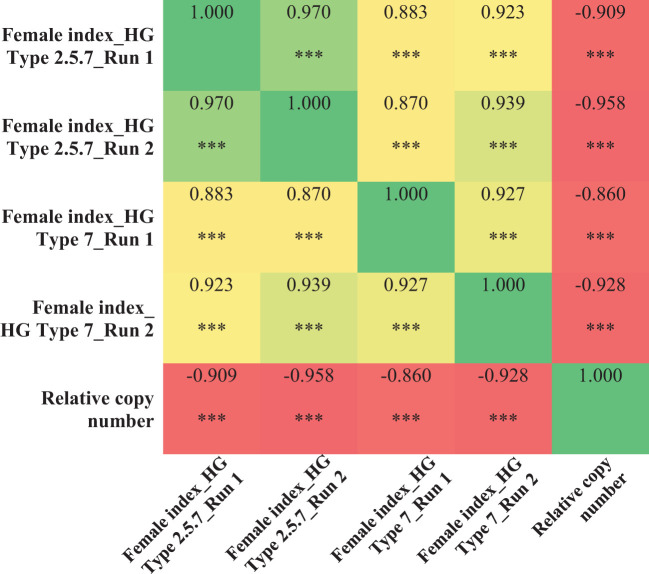
Correlation heat map showing the relationship between the copy numbers at the *Rhg1* locus, determined by qPCR assay, and the female index values obtained from SCN (HG type 7 and 2.5.7) screening experiments across two experimental runs. *** indicates significant at *P*< 0.001.

**Figure 6:**
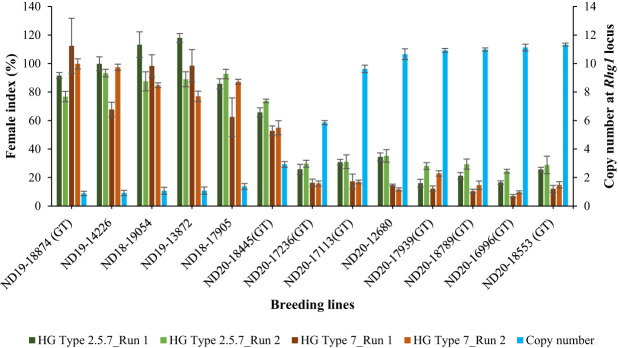
Relationship between female index (%) and copy number at *Rhg1* locus of 13 selected breeding lines. The primary y-axis represents the female index (± standard deviation), while the secondary y-axis (blue bars) represents the relative copy number (± 95% confidence interval) at the *Rhg1* locus. Female index values of the breeding lines screened for HG Type 2.5.7 are indicated by dark green and light green bars for run 1 and run 2, respectively, while those for HG Type 7 are represented by dark brown and light brown bars for run 1 and run 2, respectively. ‘Barnes’ was used as a susceptible check for calculating the female index, and ‘Williams 82’ was used as a reference for copy number determination.

Using the validated qPCR assay, the relative copy number of all the 100 breeding lines were determined. Copy number at the *Rhg1* locus ranged from 1 to 11 among the breeding lines. Specifically, 20 lines had 11 copies, 21 lines had 10 copies, one line had 6 copies, one line had 3 copies, and the remaining 57 lines had only one copy of the *Rhg1* repeat. The breeding lines having a high copy number (≥ 9) were consistently resistant (R) or moderately resistant (MR) to population S2, while the lines with a low copy number (≤ 3) were either moderately susceptible (MS) or susceptible (S) in both experimental runs. For population S1, among the 41 breeding lines with copy number ≥ 9, 31 lines were moderately resistant (MR) and 10 lines were moderately susceptible (MS) in run 1, while 24 lines were moderately resistant (MR) and the remaining 18 lines were moderately susceptible (MS) in run 2. Although, the female index for all 41 breeding lines didn’t exceed 40% in both runs of experiment for population S1. Strong negative correlations were observed between the copy number and female index for population S1 (*r*=-0.909 for run 1 and *r*=-0.958 for run 2) and for population S2 (*r*= -0.860 for run 1 and *r*=-0.928 for run 2) (**Figure 5**). Additionally, the single line with 6 copies of *Rhg1* repeats showed moderate resistance to both SCN populations S1 and S2, with female index values of 25.8% and 16.3% in run 1, and 29.7% and 15.8% in run2, respectively (**Figure 6**).

## 4 Discussion

In the majority of soybean-producing states within the United States, there has been a discernible escalation in the virulence of SCN populations, coupled with an enhanced reproductive capability specifically on the SCN-resistant PI 88788 line (Mitchum et al., 2007; Hershman et al., 2008; Niblack et al., 2008; Chen et al., 2010; Faghihi et al., 2010; Acharya et al., 2016). The diversity of SCN in field populations reflects the nematode’s adaptability and variability across different environmental conditions and cropping systems, driven by its ability to overcome resistance genes in soybean and give rise to HG types capable of parasitizing specific resistant lines. The female index serves as a pivotal indicator for evaluating the reproductive potential of SCN populations within a specific soybean line (Beeman et al., 2016; Tylka, 2016). This study compared the female index of two SCN populations S1 and S2 within the PI 88788 line. The average female index for population S1 was 25.4%, significantly higher than the 8.0% observed for population S2 in the HG type test. Screening of 100 breeding lines against two SCN populations S1 and S2 revealed differences in the resistance responses of the breeding lines based on the HG type. A greater number of breeding lines tested were resistant and moderately resistant to population S2 compared to S1. This disparity can be attributed to the varying genetic diversity of distinct SCN populations, each demonstrating distinct capabilities in parasitizing soybean lines (Koenning, 2004; Beeman et al., 2016; Tylka, 2016). Contrary to our findings, Acharya et al. (2017) observed a similar resistance response for HG type 7 and HG type 2.5.7. The difference in the findings of these two studies might be due to variations in the pathogen virulence, genetic background of the soybean lines used or different other environmental factors. In this study, most of the lines screened for population S1 were susceptible in both runs, suggesting that this SCN population is more virulent than S2 and may overcome resistance due to factors related to both pathogen virulence and genetics of the breeding lines, leading to increased number of white females on the root system (Concibido et al., 2004; Niblack et al., 2008). Similar to our finding, Acharya and Yan (2022) reported that the majority of the 149 screened soybean accessions were susceptible to HG type 2.5.7. Some breeding lines tested showed different resistance categories for the same SCN population in two runs of experiment. Variation in resistance categories for the same HG type across experimental runs may result from differences in environmental conditions, such as watering amounts and inoculum variations, all of which can influence SCN infestation and plant resistance responses.

CNVs constitute a crucial facet of genetic diversity that has a significant impact on the regulation of gene expression, the manifestation of phenotypic traits, and the process of adaptation (Shlien and Malkin, 2009; Anderson et al., 2014; Lye and Purugganan, 2019). In soybeans, CNVs at the *Rhg1* locus have been studied using fiber-fluorescence *in situ* hybridization (fiber-FISH) (Cook et al., 2012, 2014). However, fiber-FISH is expensive, technically challenging, and has low resolution making it difficult to detect small events and precise breakpoints. Additionally, this method has low throughput, requires manual curation, and only detects specific abnormalities (Soong et al., 2020). Whole genome sequencing (WGS) is an alternative approach that estimates CNVs through read depth analysis by comparing reads aligned to the tandemly repeated *Rhg1* region with those aligned to non-repeated regions outside the repeat region (Cook et al., 2012, 2014; Lee et al., 2015, 2016; Patil et al., 2019). However, this method is not inherently quantitative, and factors such as short reads, variability in sequencing quality and coverage across soybean accessions, and the structurally complex nature of the *Rhg1* locus characterized by structural variations and repetitive sequences, complicate assembly, alignment and analysis (Lee et al., 2015; Gabrielaite et al., 2021; Coutelier et al., 2022). Additionally, phasing analysis of the *Rhg1* repeats is labor-intensive, requiring precise identification of distinct repeat subtypes, manual reconstruction of repeat units, and validation with fosmid clones, further adding to the complexity of the process (Lee et al., 2015). Quantitative real-time PCR (qPCR) offers a more efficient alternative for detecting CNVs at the targeted *Rhg1* locus (Lee et al., 2016; Liu et al., 2017; Huang et al., 2021). Unlike fiber-FISH and whole genome sequencing, qPCR is quantitative, and measures the accumulation of PCR products in real-time by correlating the quantification cycle (Cq) value with the initial template quantity, with lower Cq values indicating higher target DNA concentrations, providing precise estimates of CNVs (Ginzinger, 2002; Li et al., 2004). The use of standard curves and internal controls for normalization enhances its accuracy and allows for direct comparisons between samples. Compared to sequencing methods, qPCR also offers higher throughput, lower cost, and simpler analysis, making it an ideal tool for CNVs studies.

The SYBR Green-based real-time qPCR assay was developed in this study, using the primers designed in the previous study reported by Lee et al. (2015), targeting the *Glyma18g02590* gene at *Rhg1* locus. The assay was optimized for primer concentration, annealing temperature and DNA template amount to precisely quantify the copy number at the *Rhg1* locus in the 100 breeding lines. The SYBR Green-based qPCR diagnostic system was adopted in this study because it is relatively cheaper than other probe-based methods without compromising accuracy (Andersen et al., 2006; Tran et al., 2023). However, this diagnostic system does require optimization of conditions for increased reaction efficiency (Monis et al., 2005). The qPCR assay used in this study has been optimized and validated using twelve soybean accessions with known copy number at *Rhg1* locus determined by whole-genome sequencing in Lee et al. (2015). A high degree of correlation (*r*=0.994) between the copy number detected by the qPCR assay in this study and whole genome sequencing in Lee et al. (2015) validates the qPCR assay. The target gene, *Glyma18g02590* encodes an α-SNAP protein and known to involve in resistance against SCN (Cook et al., 2012). Studies on *Rhg1*-encoded α-SNAP proteins have shown that they disrupt vesicle trafficking, cause cytotoxicity, and deplete SNARE-recycling 20S complexes, ultimately impacting SCN resistance in soybeans (Bayless et al., 2016). The *Hsp* gene used as an internal control in this study has been previously used in some studies (Li et al., 2009; Xing et al., 2010; Lee et al., 2015). This expression of this gene is relatively stable across all samples and normalizes the gene expression, accounting for any differences in the initial concentration of the DNA when comparing different DNA samples in the qPCR reaction (Galiveti et al., 2010). Williams 82 with a single copy of the *Rhg1* repeat (Cook et al., 2012) was used to calibrate each ΔCq value, which is a measure of the copy number of the target segment relative to the internal control segment. The upper bound (RCN_max_) and lower bound (RCN_min_) of the relative copy number calculated in this study helps to estimate the probable range of the copy number at 95% confidence interval (Weaver et al., 2010). The qPCR amplification efficiency was calculated using the slope of the standard curve equation to assess the assay performance and ensure accurate quantification of target DNA, while melt curve was analyzed to assess the specificity and purity of the PCR products. The qPCR assay efficiency was 94.36% for the target *Glyma18g02590* gene and 101.39% for the internal control *Hsp* gene, which is within the optimal efficiency acceptance range of 90–110% (Mukherjee et al., 2023), indicating the primers were applicable to the qPCR assay. A well-defined, symmetric peak with a single sharp peak with single melting peak at 82°C and 76.5°C for the target *Glyma18g02590* gene and reference *Hsp* gene, respectively were observed, indicating that only one specific product was amplified (Harshitha and Arunraj, 2021). These analyses demonstrate the utilization of an optimized qPCR assay for the quantification of copy number variations at the *Rhg1* locus in our study.

PI 88788 is a predominant donor source for breeding soybean cultivars in the United States with resistance to SCN, with the pivotal gene responsible for this resistance being *rhg1*-b (Cook et al., 2012). Resistance to SCN controlled by *Rhg1* varies significantly, even among lines originating the same resistance source, due to its complex genetic system involving second-site modifier loci (Concibido et al., 2004; Niblack et al., 2009; Lee et al., 2016). Repeat instability at *Rhg1* contributes to this variability, allowing selection for higher copy numbers within the PI88788-derived breeding lines to enhance SCN resistance. Patil et al. (2019) reported that effective resistance against SCN populations with no or less ability to reproduce in PI 88788 and its derivative soybean lines featuring the *Rhg1*-b locus, requires a minimum of 5.6 *rhg1*-b gene, based on their study focusing on haplotype analysis using different SCN populations and the interaction of *Rhg1* and *Rhg4*. Higher number of copies within the *Rhg1* locus has been linked to increased resistance against SCN (Cook et al., 2014; Lee et al., 2016; Yu et al., 2016; Patil et al., 2019; Han et al., 2020; St-Amour et al., 2020). In a study reported by Cook et al. (2014), two distinct groups of *Rhg1* repeats were found in 41 diverse soybean accessions: a high copy number group with 7-10 repeats and a low copy number group with ≤ 3 repeats. Additionally, Lee et al. (2015) examined soybean accessions and discovered that the copy number variation at the *Rhg1* locus spans from 1 to 10, indicating a broad range of genetic diversity in soybeans regarding this specific gene region. While numerous efforts have been made to identify CNVs at the *Rhg1* locus across a large number of soybean accessions, there remains a noticeable gap in research regarding the extent to which CNVs differ across the breeding lines. In our study, copy number ranged from 1 to 11 among the 100 breeding lines. Moreover, a highly significant negative correlation was observed between copy number and female indices, suggesting that an increased copy number at *Rhg1* is indicative of greater resistance to SCN. We found that the breeding lines with copy number ≥ 9 were either resistant or moderately resistant to SCN population HG type 7 (S2) with female index less than 30%, and most of those lines were moderately resistant to HG type 2.5.7 (S1) (FI<30%), with few being moderately susceptible but with female index less than 40% (**Figure 6**). This result highlights the strong association between high copy numbers and increased resistance to HG type 7, though this relationship may vary for HG type 2.5.7. Selecting breeding lines with a higher copy number is most effective against HG type 7 and can also improve resistance to HG type 2.5.7, but to a lesser extent than for HG type 7. However, incorporating *Rhg4* from Peking with high-copy *Rhg1* from PI 88788 could broaden resistance to HG type 2.5.7 (Patil et al., 2019). Additionally, stacking *Rhg1* variants from both Peking and PI 88788 in a single line could improve resistance durability and help prevent the development of virulent SCN populations (Cook et al., 2014). The breeding line, ND20-17236(GT), characterized by 6 copies of *Rhg1* repeats in this study, showed moderate resistance to both SCN populations S1 and S2. This observation may also suggest the potential presence of an additional gene expressed at the *Rhg1* locus. Given the focus of this study on *Glyma18g02590*, further investigation into potential co-expression or genetic interactions of resistance genes in this line could provide insights into the mechanisms underlying resistance in this breeding line. The integration of both phenotypic and molecular methodologies in this study has facilitated the exploration of potential correlations between the resistance response and CNVs at the *Rhg1* locus. The findings of these two approaches appear to be mutually corroborative. Traditional SCN screening experiments are often labor-intensive and time-consuming (Lopez-Nicora et al., 2012). However, the qPCR assay optimized and used in this study is fast, simple, cost-effective, and holds promise for assessing diverse soybean lines for potential resistance to SCN.

## 5 Conclusion

SCN remains a critical threat to soybean production, resulting in substantial yield losses. The diverse populations of SCN across different fields necessitate management strategies that involve identifying soybean lines resistant to specific HG types of the nematode. Resistance varies among soybean lines depending on the HG types, with increased susceptibility linked to the nematode ‘s ability to reproduce in PI 88788. CNVs at the *Rhg1* locus play a crucial role in determining resistance levels in soybean lines. This study optimized the qPCR assay and used this quantitative PCR approach to quantify the CNVs at the *Rhg1* locus. The results of this study showed that increased copy number at *Rhg1* is associated with enhanced resistance to SCN with reduced ability to reproduce in the PI88788 source of resistance. However, resistance against HG type 2.5.7, a more virulent SCN population, was less effective with an enhanced ability of the nematode to reproduce in PI 88788, despite higher copy numbers still providing some level of resistance. These findings suggest that while high copy numbers at *Rhg1* contribute significantly to SCN resistance, their efficacy can vary depending on the virulence of the SCN population. The information generated in this study would help understand the relationship of copy number variations and resistance levels to different virulence phenotypes of SCN, facilitate the identification and selection of soybean lines with high copy numbers at *Rhg1*, and speed up the *Rhg1* locus genotyping for diverse soybean lines to further improve SCN resistance and reduce the impact of this devastating nematode pest in soybean.

## Data Availability

The datasets presented in this study can be found in online repositories. The names of the repository/repositories and accession number(s) can be found in the article/**Supplementary Material**.
