# Supplementary material for: Copy number variations at the Rhg1 locus and their relationship with resistance to soybean cyst nematode (Heterodera glycines)
Source: Front Plant Sci. 2024 Dec 18;15:1504932. doi: 10.3389/fpls.2024.1504932 (PMC11736665; doi:10.3389/fpls.2024.1504932)
Supplement: Supplementary file 1 [file DataSheet1.docx]

Supplementary Table 1: Analysis of variance for the effects of breeding line, run, HG type and their interactions on the numbers of white females formed on the roots of the breeding lines in phenotypic screening for SCN resistance.

| **Effect** | **Degree of freedom** | **Sum of square** | **F value** | **Probability>F** |
| --- | --- | --- | --- | --- |
| Breeding line | 99 | 11288881 | 607.21 | <0.0001 |
| Run | 1 | 2906343 | 15476.52 | <0.0001 |
| HG type | 1 | 715124 | 3808.096 | <0.0001 |
| Breeding line × Run | 99 | 2705782 | 145.54 | <0.0001 |
| Breeding line × HG type | 99 | 504300 | 27.13 | <0.0001 |
| HG type × Run | 1 | 9526 | 50.73 | <0.0001 |
| Breeding line × Run × HG type | 99 | 1235010 | 66.43 | <0.0001 |

Supplementary Table 2: Female Index in four plant introduction lines (PI 548408, PI 88788, PI 209332 and PI 54316), that were included as checks in the screening of 100 breeding lines.

| **Plant Introductions (PIs)** |  | **Female Index (%)** | | | | |
| --- | --- | --- | --- | --- | --- | --- |
|  | **S1** | | |  | **S2** | |
|  | **Run 1** | | **Run 2** |  | **Run 1** | **Run 2** |
| PI 548408 (Peking) | 3.7 | | 3.0 |  | 3.4 | 2.1 |
| PI 88788 | 26.8 | | 24.9 |  | 9.3 | 8.6 |
| PI 209332 | 25.0 | | 22.3 |  | 9.3 | 9.2 |
| PI 54316 | 17.4 | | 15.9 |  | 29.0 | 33.3 |

Barnes was used as a susceptible check in both runs for both SCN populations. The average number of white females in the susceptible checks were 303 and 416 for population S2, and 279 and 431 for population S1 for run1 and run 2, respectively.

Supplementary Table 3: Summary of female index (FI), resistance response (RR) and copy number at *Rhg1* locus determined by qPCR assay for 100 breeding lines.

| **Breeding Lines** | **S1** | | | | **S2** | | | | **Copy number** |
| --- | --- | --- | --- | --- | --- | --- | --- | --- | --- |
|  | **Run 1** | | **Run 2** | | **Run 1** | | **Run 2** | |  |
|  | **FI^a^** | **RR^b^** | **FI** | **RR** | **FI** | **RR** | **FI** | **RR** |  |
| ND18-16823 | 101.0 | S | 87.4 | S | 51.1 | MS | 62.1 | S | 1 |
| ND18-17021 | 82.6 | S | 86.0 | S | 46.5 | MS | 56.6 | MS | 1 |
| ND18-17201 | 89.1 | S | 87.6 | S | 64.6 | S | 64.8 | S | 1 |
| ND18-17666 | 29.9 | MR | 27.4 | MR | 15.3 | MR | 12.0 | MR | 11 |
| ND18-17905 | 85.8 | S | 92.6 | S | 62.4 | S | 87.2 | S | 1 |
| ND18-18369 | 85.0 | S | 84.9 | S | 78.8 | S | 82.7 | S | 1 |
| ND18-19054 | 113.2 | S | 87.5 | S | 98.2 | S | 84.7 | S | 1 |
| ND18-25147 (GT) | 95.1 | S | 88.2 | S | 53.5 | MS | 66.8 | S | 1 |
| ND19-13465 | 86.3 | S | 85.4 | S | 45.4 | MS | 66.6 | S | 1 |
| ND19-13737 | 92.5 | S | 88.7 | S | 46.7 | MS | 65.9 | S | 1 |
| ND19-13759 | 92.5 | S | 88.2 | S | 80.3 | S | 95.3 | S | 1 |
| ND19-13872 | 117.9 | S | 88.9 | S | 98.5 | S | 77.0 | S | 1 |
| ND19-13873 | 104.4 | S | 87.7 | S | 82.7 | S | 81.5 | S | 1 |
| ND19-13905 | 93.2 | S | 90.2 | S | 41.6 | MS | 66.0 | S | 1 |
| ND19-14086 | 86.9 | S | 89.3 | S | 71.9 | S | 85.2 | S | 1 |
| ND19-14226 | 99.8 | S | 93.2 | S | 67.7 | S | 97.3 | S | 1 |
| ND19-18020 (GT) | 22.5 | MR | 29.5 | MR | 24.4 | MR | 18.6 | MR | 11 |
| ND19-18189 (GT) | 98.3 | S | 87.0 | S | 91.6 | S | 79.7 | S | 1 |
| ND19-18200 (GT) | 91.1 | S | 87.6 | S | 143.8 | S | 82.7 | S | 1 |
| ND19-18258 (GT) | 99.3 | S | 88.5 | S | 46.5 | MS | 46.8 | MS | 1 |
| ND19-18438 (GT) | 85.5 | S | 74.0 | S | 96.7 | S | 90.0 | S | 1 |
| ND19-18874 (GT) | 91.5 | S | 76.8 | S | 112.4 | S | 99.7 | S | 1 |
| ND19-19257 (GT) | 96.4 | S | 79.5 | S | 77.9 | S | 102.7 | S | 1 |
| ND19-19372 (GT) | 84.8 | S | 74.1 | S | 50.7 | MS | 64.0 | S | 1 |
| ND20-11544 | 64.7 | S | 68.0 | S | 51.2 | MS | 54.0 | MS | 1 |
| ND20-11644 | 31.7 | MS | 33.5 | MS | 12.0 | MR | 22.4 | MR | 10 |
| ND20-11822 | 26.8 | MR | 28.2 | MR | 17.2 | MR | 12.2 | MR | 11 |
| ND20-12420 | 70.3 | S | 74.2 | S | 63.5 | S | 64.6 | S | 1 |
| ND20-12680 | 34.5 | MS | 35.1 | MS | 14.7 | MR | 11.6 | MR | 11 |
| ND20-12722 | 75.2 | S | 74.9 | S | 50.2 | MS | 56.5 | MS | 1 |
| ND20-12884 | 63.2 | S | 62.6 | S | 56.2 | MS | 62.2 | S | 1 |
| ND20-13062 | 68.6 | S | 70.6 | S | 54.2 | MS | 63.5 | S | 1 |
| ND20-13070 | 68.1 | S | 70.9 | S | 68.1 | S | 65.1 | S | 1 |
| ND20-13836 | 67.5 | S | 72.4 | S | 50.0 | MS | 52.4 | MS | 1 |
| ND20-14125 | 26.5 | MR | 26.9 | MR | 20.4 | MR | 17.4 | MR | 11 |
| ND20-14431 | 29.3 | MR | 31.3 | MS | 19.9 | MR | 21.6 | MR | 10 |
| ND20-14446 | 29.8 | MR | 34.1 | MS | 19.5 | MR | 26.4 | MR | 10 |
| ND20-14452 | 92.8 | S | 78.7 | S | 61.1 | S | 57.4 | MS | 1 |
| ND20-14522 | 72.6 | S | 74.2 | S | 71.8 | S | 67.1 | S | 1 |
| ND20-14650 | 62.6 | S | 64.4 | S | 56.2 | MS | 58.8 | MS | 1 |
| ND20-14651 | 87.1 | S | 78.8 | S | 68.4 | S | 64.0 | S | 1 |
| ND20-14806 | 31.4 | MS | 31.9 | MS | 18.3 | MR | 23.5 | MR | 10 |
| ND20-14829 | 69.4 | S | 70.0 | S | 61.2 | S | 61.4 | S | 1 |
| ND20-14986 | 28.2 | MR | 30.8 | MS | 17.3 | MR | 17.5 | MR | 10 |
| ND20-15035 | 67.8 | S | 65.0 | S | 63.3 | S | 71.9 | S | 1 |
| ND20-16462(GT) | 25.1 | MR | 28.2 | MR | 17.0 | MR | 21.9 | MR | 10 |
| ND20-16636(GT) | 28.4 | MR | 28.7 | MR | 19.5 | MR | 16.4 | MR | 11 |
| ND20-16644(GT) | 31.5 | MS | 34.3 | MS | 15.1 | MR | 20.7 | MR | 10 |
| ND20-16667(GT) | 91.4 | S | 74.3 | S | 62.1 | S | 68.7 | S | 1 |
| ND20-16891(GT) | 69.2 | S | 72.9 | S | 60.5 | S | 65.5 | S | 1 |
| ND20-16969(GT) | 34.0 | MS | 33.7 | MS | 19.7 | MR | 25.7 | MR | 11 |
| ND20-16996(GT) | 16.5 | MR | 24.4 | MR | 7.0 | R | 9.7 | R | 11 |
| ND20-17039(GT) | 38.1 | MS | 34.3 | MS | 19.8 | MR | 25.1 | MR | 10 |
| ND20-17081(GT) | 67.3 | S | 70.5 | S | 60.1 | S | 71.4 | S | 1 |
| ND20-17086(GT) | 27.3 | MR | 29.6 | MR | 18.0 | MR | 19.7 | MR | 10 |
| ND20-17113(GT) | 30.8 | MS | 31.0 | MS | 17.2 | MR | 16.9 | MR | 10 |
| ND20-17115(GT) | 28.6 | MR | 27.2 | MR | 12.4 | MR | 18.4 | MR | 11 |
| ND20-17236(GT) | 25.8 | MR | 29.7 | MR | 16.3 | MR | 15.8 | MR | 6 |
| ND20-17237(GT) | 82.6 | S | 73.8 | S | 60.3 | S | 67.4 | S | 1 |
| ND20-17262(GT) | 43.5 | MS | 60.6 | S | 44.5 | MS | 57.0 | MS | 1 |
| ND20-17433(GT) | 32.3 | MS | 34.4 | MS | 17.6 | MR | 13.8 | MR | 10 |
| ND20-17443(GT) | 26.3 | MR | 27.8 | MR | 16.3 | MR | 24.9 | MR | 11 |
| ND20-17676(GT) | 71.3 | S | 73.0 | S | 55.6 | MS | 66.0 | S | 1 |
| ND20-17682(GT) | 85.7 | S | 73.4 | S | 74.4 | S | 68.1 | S | 1 |
| ND20-17731(GT) | 20.4 | MR | 26.6 | MR | 17.5 | MR | 19.6 | MR | 11 |
| ND20-17744(GT) | 32.6 | MS | 33.7 | MS | 22.0 | MR | 23.0 | MR | 10 |
| ND20-17746(GT) | 83.9 | S | 74.3 | S | 79.9 | S | 85.9 | S | 1 |
| ND20-17748(GT) | 28.1 | MR | 29.2 | MR | 15.7 | MR | 17.2 | MR | 10 |
| ND20-17783 (GT) | 24.5 | MR | 28.6 | MR | 16.0 | MR | 15.7 | MR | 11 |
| ND20-17799(GT) | 28.5 | MR | 29.4 | MR | 15.2 | MR | 17.0 | MR | 10 |
| ND20-17939(GT) | 16.1 | MR | 28.1 | MR | 12.2 | MR | 22.9 | MR | 11 |
| ND20-17978(GT) | 66.6 | S | 72.5 | S | 45.6 | MS | 60.2 | S | 1 |
| ND20-18152(GT) | 58.7 | MS | 71.0 | S | 53.9 | MS | 67.9 | S | 1 |
| ND20-18222(GT) | 70.3 | S | 71.2 | S | 61.9 | S | 66.4 | S | 1 |
| ND20-18244(GT) | 66.2 | S | 71.0 | S | 59.7 | MS | 68.9 | S | 1 |
| ND20-18339(GT) | 67.1 | S | 68.3 | S | 64.3 | S | 63.1 | S | 1 |
| ND20-18356(GT) | 71.9 | S | 72.9 | S | 56.2 | MS | 67.4 | S | 1 |
| ND20-18445(GT) | 65.7 | S | 73.7 | S | 52.7 | MS | 54.9 | MS | 3 |
| ND20-18448(GT) | 74.4 | S | 74.0 | S | 61.1 | S | 69.7 | S | 1 |
| ND20-18455 (GT) | 71.1 | S | 70.4 | S | 60.1 | S | 56.3 | MS | 1 |
| ND20-18458(GT) | 70.3 | S | 72.6 | S | 54.3 | MS | 64.1 | S | 1 |
| ND20-18460(GT) | 71.8 | S | 75.5 | S | 64.0 | S | 61.7 | S | 1 |
| ND20-18553 (GT) | 25.6 | MR | 28.8 | MR | 12.1 | MR | 14.8 | MR | 11 |
| ND20-18556(GT) | 29.2 | MR | 32.2 | MS | 19.4 | MR | 19.9 | MR | 10 |
| ND20-18557(GT) | 24.5 | MR | 29.3 | MR | 19.4 | MR | 16.0 | MR | 11 |
| ND20-18565(GT) | 27.3 | MR | 28.3 | MR | 15.8 | MR | 17.4 | MR | 10 |
| ND20-18674(GT) | 29.9 | MR | 31.6 | MS | 20.3 | MR | 17.4 | MR | 10 |
| ND20-18729(GT) | 35.2 | MS | 35.5 | MS | 16.7 | MR | 19.8 | MR | 10 |
| ND20-18732(GT) | 28.7 | MR | 30.7 | MS | 13.7 | MR | 22.1 | MR | 11 |
| ND20-18786(GT) | 29.5 | MR | 28.5 | MR | 18.5 | MR | 23.2 | MR | 10 |
| ND20-18789(GT) | 21.2 | MR | 29.4 | MR | 10.5 | MR | 14.7 | MR | 11 |
| ND20-18829(GT) | 71.2 | S | 73.1 | S | 68.2 | S | 66.4 | S | 1 |
| ND20-18841(GT) | 29.3 | MR | 31.2 | MS | 12.2 | MR | 21.0 | MR | 11 |
| ND20-18842(GT) | 26.6 | MR | 30.4 | MS | 14.3 | MR | 20.0 | MR | 11 |
| ND20-18846(GT) | 67.0 | S | 72.1 | S | 60.4 | S | 67.3 | S | 1 |
| ND20-18854(GT) | 27.8 | MR | 28.6 | MR | 11.8 | MR | 16.9 | MR | 10 |
| ND20-18871(GT) | 62.2 | S | 73.3 | S | 49.0 | MS | 53.8 | MS | 1 |
| ND20-18872(GT) | 16.1 | MR | 26.9 | MR | 12.3 | MR | 16.6 | MR | 10 |
| ND20-18874(GT) | 67.6 | S | 75.7 | S | 60.8 | S | 68.3 | S | 1 |
| ND20-18905(GT) | 27.5 | MR | 29.6 | MR | 11.7 | MR | 17.7 | MR | 11 |

Barnes was used as a susceptible check in both runs for both HG types. The average number of white females in the susceptible checks were 303 and 416 for population S2, and 279 and 431 for population S1 for run1 and run 2 respectively.

FI^a^ (Female index): mean number of white females produced on the tested soybean line / mean number of white females on the susceptible check Barnes x 100%.

RR^b^ (Resistance response): resistant (R) (FI<10%), moderately resistant (MR) (FI= 10 to <30%), moderately susceptible (MS) (FI= 30 to <60%), or susceptible (S) (FI ≥ 60%).

**B**

**A**


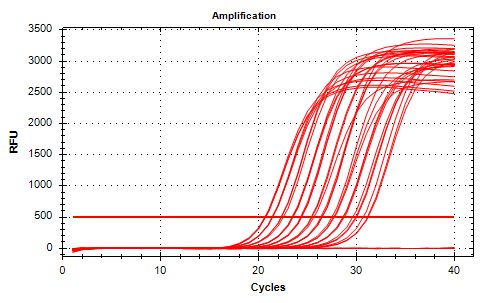

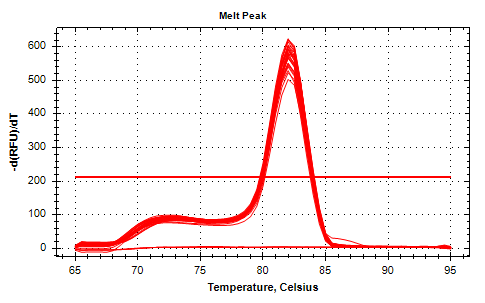


**C**

**D**


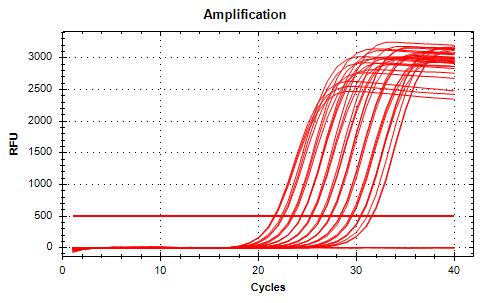

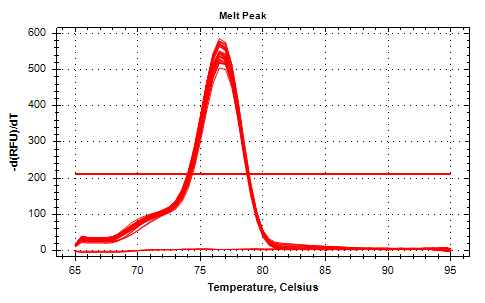


Supplementary Figure S1: Amplification and melting curve analysis from qPCR assays using serially diluted genomic DNA extracted from leaves of Williams 82, conducted with 200 nM primer concentration, 60°C annealing temperature, and 1.5 µL of DNA template. **(A)** Amplification curve of target gene at *Rhg1* locus- *Glyma18g02590.* **(B)** Melting curve profiles of target gene at *Rhg1* locus- *Glyma18g02590*-specific amplicons with melting temperature at 82°C. **(C)** Amplification curve of endogenous control- *Hsp* gene. **(D)** Melting curve profiles of endogenous control- *Hsp* -specific amplicons with melting temperature at 76.5°C.
